# NOVEL FINDINGS AND LESSONS LEARNED FROM CROSS-NATIONAL LONGITUDINAL RESEARCH ON MIDLIFE DEVELOPMENT AND AGING

**DOI:** 10.1093/geroni/igae098.2196

**Published:** 2024-12-31

**Authors:** Frank Infurna, Denis Gerstorf, Lisbeth Nielsen

**Affiliations:** Chair; Co-Chair; Discussant

## Abstract

The goal of this symposium is to bring together a collection of papers that shed light on opportunities gained and challenges encountered in cross-national comparisons of development in midlife and aging and lessons learned. Infurna and colleagues used longitudinal panel data from 16 nations to examine historical changes in midlife pain and report historical increases in pain in the U.S., and Continental and Nordic Europe, whereas Mediterranean Europe, South Korea, and Mexico experienced historical declines. Wettstein and colleagues studied historical changes in midlife memory and explanatory factors in longitudinal panel data from 16 nations; the U.S. was the only nation/region to exhibit historical declines in memory, whereas historical improvements were observed in the European nations. Women, higher educational attainment, better grip strength, and fewer chronic diseases were associated with better memory across cohorts. Steptoe and Brocklebank used data from England and China to examine how physical activity and sleep patterns relate to cognitive decline and highlight challenges related to data collection and instrumentation and approaches to carry out robust cross-national comparisons. Wong provides an overview of research on cross-national comparisons using the Mexico Health and Aging Study by illustrating various examples and discussing gaps in knowledge pertaining to how low and middle-income nations could be supported by cross-national data. The discussion by Nielsen will integrate the four papers by highlighting novel findings that can arise from conducting cross-national research (e.g., historical change, comparisons of explanatory factors) and insights gained through the increasing availability of comparable data that can be harmonized.

